# Prognostic significance of triglyceride-glucose index in acute coronary syndrome patients without standard modifiable cardiovascular risk factors

**DOI:** 10.1186/s12933-024-02345-5

**Published:** 2024-07-23

**Authors:** Xiaoming Zhang, Yu Du, Tianhao Zhang, Zehao Zhao, Qianyun Guo, Xiaoteng Ma, Dongmei Shi, Yujie Zhou

**Affiliations:** Department of Cardiology, Beijing Anzhen Hospital, Beijing Institute of Heart Lung and Blood Vessel Disease, Beijing Key Laboratory of Precision Medicine of Coronary Atherosclerotic Disease, Clinical Center for Coronary Heart Disease, Capital Medical University, Beijing, 100029 China

**Keywords:** Triglyceride glucose index, Standard modifiable cardiovascular risk factors, Acute coronary syndrome, Prognosis

## Abstract

**Background:**

A significant percentage of patients with acute coronary syndrome (ACS) without standard modifiable cardiovascular risk factors (SMuRFs) are being identified. Nonetheless, the prognostic influence of the TyG index on adverse events in this type of patient remains unexplored. The aim of this study was to assess the prognostic value of the TyG index among ACS patients without SMuRFs for predicting adverse outcomes.

**Methods:**

This study involved 1140 consecutive patients who were diagnosed with ACS without SMuRFs at Beijing Anzhen Hospital between May 2018 and December 2020 and underwent coronary angiography. Each patient was followed up for a period of 35 to 66 months after discharge. The objective of this study was to examine major adverse cardiac and cerebrovascular events (MACCE), which included all-cause mortality, non-fatal myocardial infarction, non-fatal ischemic stroke, as well as ischemia-driven revascularization.

**Results:**

During the median follow-up period of 48.3 months, 220 (19.3%) MACCE events occurred. The average age of the participants was 59.55 ± 10.98 years, and the average TyG index was 8.67 ± 0.53. In the fully adjusted model, when considering the TyG index as either a continuous/categorical variable, significant associations with adverse outcomes were observed. Specifically, for each 1 standard deviation increase in the TyG index within the highest TyG index group, there was a hazard ratio (HR) of 1.245 (95% confidence interval CI 1.030, 1.504) for MACCE and 1.303 (95% CI 1.026, 1.653) for ischemia-driven revascularization (both P < 0.05), when the TyG index was analyzed as a continuous variable. Similarly, when the TyG index was examined as a categorical variable, the HR (95% CI) for MACCE in the highest TyG index group was 1.693 (95% CI 1.051, 2.727) (P < 0.05) in the fully adjusted model, while the HR (95% CI) for ischemia-driven revascularization was 1.855 (95% CI 0.998, 3.449) (P = 0.051). Additionally, the TyG index was found to be associated with a poor prognosis among the subgroup.

**Conclusion:**

The TyG index is correlated with poor prognosis in patients with ACS without SMuRFs, suggesting that it may be an independent predictive factor of adverse events among these individuals.

## Background

Over the past three decades, cardiovascular disease (CVD) has remained the leading cause of death worldwide. Among all CVD categories, ischemic heart disease has the highest disability-adjusted life years based on age [1]. In the field of CVD research, hypertension, diabetes, hypercholesterolemia, and smoking are standard modifiable cardiovascular risk factors (SMuRFs) [2–4]. These risk factors play a crucial role in both primary and secondary prevention strategies in modern CVD preventive medicine [5]. However, recent studies have revealed a subset of acute coronary syndrome (ACS) patients who, at the onset of their illness, do not exhibit any of the mentioned risk factors and are thus categorized as SMuRF-less ACS patients [3]. It has been observed that the proportion of such ACS patients is increasing [6–8], and compared to patients with at least one risk factor, those with worse prognosis have higher mortality rates [3, 7, 9–12].

The development of coronary artery atherosclerosis has been closely linked to insulin resistance (IR) [13–15], even though it is not recognized as a standard risk factor [16]. The absence of standard cardiovascular risk factors in SMuRF-less ACS patients underscores the importance of identifying potential nonstandard risk factors for their condition, which is pivotal for risk assessment and therapeutic guidance. A reliable indicator of IR status is the triglyceride-glucose (TyG) index [17–19], which only requires the patient’s triglyceride (TG) and fasting blood glucose (FBG) levels. Its high accessibility and cost-effectiveness make it an attractive option for assessing IR levels. Several studies have demonstrated the predictive ability of the TyG index for all-cause mortality, myocardial infarction (MI), and other adverse cardiovascular events [20–23]. However, the relationship of the TyG index with adverse cardiovascular events among SMuRF-less ACS patients remains unexplored. Therefore, our aim was to investigate whether the TyG index can predict adverse cardiovascular events in SMuRF-less ACS patients.

## Methods

### Study population

**Fig. 1:**
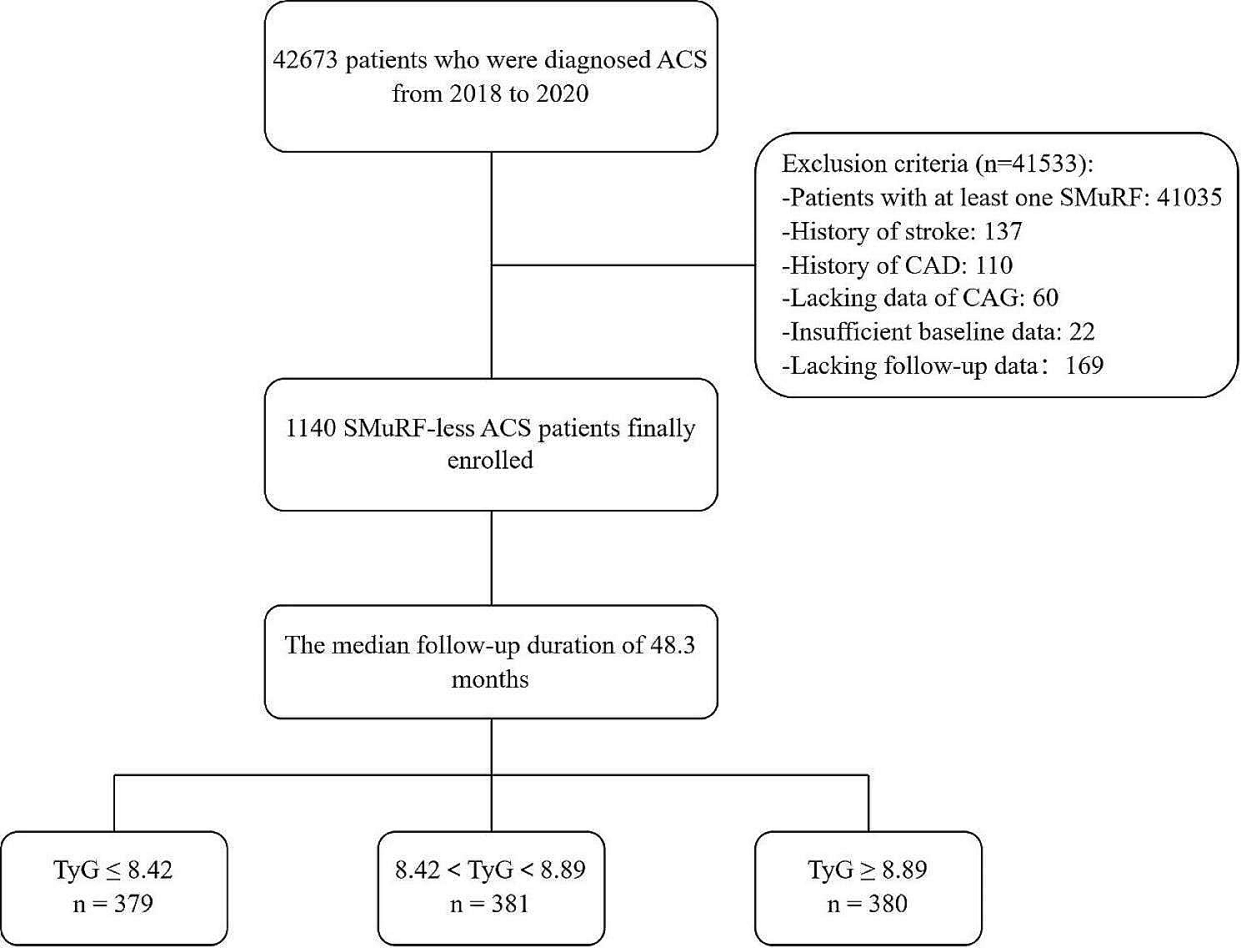
Study flow chart of inclusion and exclusion criteria of the study population. *TyG* triglyceride glucose index, *ACS* acute coronary syndrome, *SMuRFs* standard modifiable cardiovascular risk factors, *CAG* coronary angiography, *CAD* coronary artery disease

A single-center, observational, retrospective cohort study was conducted, involving 42,673 patients who were diagnosed with ACS at the Beijing Anzhen Hospital Affiliated to Capital Medical University between May 2018 and December 2020. The discharge diagnosis for ACS included unstable angina pectoris (UAP), non-ST-segment elevation myocardial infarction (NSTEMI), as well as ST-segment elevation myocardial infarction (STEMI). After excluding patients with standard modifiable cardiovascular risk factors for ACS, a total of 1391 SMuRF-less ACS patients were identified. Subsequent exclusion of patients with missing baseline data, coronary angiography (CAG) data, and those lost to follow-up led to the inclusion of 1140 individuals in the final study cohort. The detailed exclusion criteria are described in Fig. 1.

SMuRFs included hypertension, diabetes, hypercholesterolemia, and smoking [2, 4]. Patients with hypertension were defined as those with a previous diagnosis or use of hypertension medications, or having hypertension listed in the medical records as the secondary discharge diagnosis (based on a mean systolic blood pressure of ≥ 140 mmHg or diastolic blood pressure of ≥ 90 mmHg recorded from at least 2 readings obtained on separate days). Diabetes referred to a previous diagnosis of diabetes, previous administration of diabetes medications, glycated hemoglobin A1c (HbA1c) concentration ≥ 6.5% during this admission, or having diabetes listed in the medical records as the secondary discharge diagnosis. An individual with hypercholesterolemia during the index admission was defined as having a prior medical diagnosis of hypercholesterolemia, receiving previous or ongoing treatment for hypercholesterolemia, or having low-density lipoprotein cholesterol (LDL-c) ≥ 3.4 mmol/L or total cholesterol (TC) ≥ 5.2 mmol/L [24]. Smoking status included past or current smoking. Due to neurohormonal responses to acute myocardial infarction (MI), both FBG and acute phase blood pressure were not incorporated in the definitions [2]. Medical records, hospital findings, and self-reported smoking during admission served as the basis for the definition of SMuRFs.

### Data collection and definition

The data was obtained from the electronic medical records system of Beijing Anzhen Hospital. The clinical data collected included demographic data (age, sex, body mass index [BMI], blood pressure, and heart rate), clinical presentation (UAP, NSTEMI, STEMI), laboratory examinations (all patients completed serum biochemical tests, including renal and liver function, blood lipids, and electrolytes, upon admission), echocardiographic data (left ventricular ejection fraction [LVEF]), CAG data, and medication at discharge. It should be noted that the measurement of glucose level was not taken during the day of the admission, and was taken under standardized conditions. Coronary revascularization was performed according to the most recent guidelines [25, 26]. An information center at the hospital confirmed the quality of the aforementioned data.

Based on the CAG data, coronary artery stenosis was classified into single-vessel disease and multi-vessel disease (≥ 50% diameter stenosis of ≥ 2 major coronary arteries). The TyG index was determined using this formula: TyG index = Ln [fasting TG (mg/dL) × FBG (mg/dL)/2].

### Follow-up and outcomes

After hospital discharge, trained specialized clinical personnel conducted a telephone questionnaire or reviewed medical records every 6 months to identify any events. The maximum follow-up period was 66 months, with a median follow-up time of 48.3 months. The primary outcome for this study was the time until the first occurrence of major adverse cardiac and cerebrovascular events (MACCE), which encompassed all-cause mortality (cardiac or non-cardiac), non-fatal MI, non-fatal ischemic stroke, and ischemia-driven revascularization. Non-fatal MI was diagnosed based on the fourth universal definition [27], while non-fatal ischemic stroke was confirmed by clinical manifestations of neurological impairment and imaging evidence from computed tomography (CT) scans or magnetic resonance imaging. Ischemia-driven revascularization referred to the restoration of coronary artery blood flow, achieved through percutaneous coronary intervention (PCI) or coronary artery bypass grafting (CABG), in response to the patient's hospitalization due to recurrent or persistent chest pain, involving both target and non-target lesions.

### Statistical analysis

Measurement data that followed a normal distribution were reported as the mean ± standard deviation (SD). Student’s t-test or analysis of variance (ANOVA) was used if the variances were equal. Otherwise, the rank-sum test was employed. Non-normally distributed measurement data were presented as medians with interquartile ranges (IQRs). Categorical variables were expressed as percentages and compared using the chi-square test or Fisher's exact test. The log-rank test, based on the Kaplan–Meier method for describing event rates during follow-up, was employed to compare the time-to-event curves of three groups.

To observe differences among various TyG index levels, the patients were divided into three groups based on their TyG index levels: tertile 1 with TyG index ≤ 8.42, tertile 2 with 8.42 < TyG index < 8.89, and tertile 3 with TyG index ≥ 8.89. Secondly, the patients were divided into two groups depending on the presence of MACCE to investigate prognostic factors in patients with/without MACCE. The TyG index and other characteristics of patients were compared between the two groups.

The current research used univariate Cox regression analysis to examine the variables associated with ischemia-driven revascularization and MACCE. Subsequently, the study investigated the link of the TyG index with ischemia-driven revascularization and MACCE using the Cox proportional hazards model. The TyG index was assessed using both nominal and continuous variables. After adjusting for independent risk factors identified in the initial univariate Cox regression analysis and potential confounding clinical variables, three regression models were created. Model 1 was a partially adjusted model with the age, sex, and BMI controlled. Model 2 included Model 1 variables along with systolic blood pressure, heart rate, LVEF, creatinine, high-density lipoprotein cholesterol (HDL-c), LDL-c, high-sensitivity C-reactive protein (hs-CRP) and uric acid (UA). Model 3 included all variables from Model 2, as well as the left main coronary artery (LM), multi-vessel disease, operational intervention, non-ST-segment elevation acute coronary syndrome (NSTE-ACS), as well as discharge medication.

Additionally, to examine the potential impact of risk-factor control (LDL-c, blood pressure) and other variables (age, sex, BMI, multi-vessel disease status, and diagnosis) on the link of the TyG index to the prognosis, subgroup analyses were carried out. Hazard ratios (HRs), 95% confidence intervals (CIs), alongside p-values for interaction are shown on the graphs.

The statistical analysis was conducted using SPSS (IBM SPSS, version 26, Chicago, Illinois) as well as R statistical software (version 4.3.2). A two-sided P-value of < 0.05 was considered statistically significant.

## Results

### Baseline characteristics

In the study, a total of 1140 patients were included. During the median follow-up period (48.3 months), MACCE was observed among 220 (19.3%) of the 1140 patients studied, including 56 (4.9%) all-cause deaths, 14 (1.2%) strokes, 15 (1.3%) non-fatal MI, and 135 (11.8%) ischemia-driven revascularizations. Given possible fluctuation of blood glucose in the acute phase of ACS, we calculated the TyG index using glucose derived from the morning of the second day following admission for patients without acute myocardial infarction, and 8.3 days (IQR: 7.5–9.6) later after admission for patients with acute myocardial infarction. The average blood glucose level on the day of admission for all patients was 6.47 ± 1.55 mmol/l, whereas blood glucose used for the TyG index calculation was 5.78 ± 1.59 mmol/l. Table 1 presents the baseline demographics and clinical characteristics of the patients, categorized based on tertiles of the TyG index. The mean age of the participants was 59.55 ± 10.98 years, and 61.8% of the participants were male. The mean TyG index was 8.67 ± 0.53. Patients with a higher TyG index were more prone to being overweight and exhibiting elevated levels of TC, TG, LDL-c, FBG, UA, alongside hs-CRP. They were also more likely to undergo PCI intervention and take P_2_Y_12_ inhibitors (P_2_Y_12_i) (all p < 0.05). Conversely, these patients tended to be younger, had lower HDL-c levels, and were less likely to receive conservative pharmacological therapy (all p < 0.05). Additionally, an increase in the TyG index corresponded to a higher prevalence of right coronary artery (RCA) lesions and multi-vessel lesions observed during CAG (all p < 0.05). Among the intermediate TyG index group, the utilization rate of statins was found to be the lowest (p < 0.05). Table 2 divides patients into two groups based on the occurrence of MACCE during the follow-up period. Differences with statistical significance were observed between the MACCE and non-MACCE groups in terms of age, LVEF, TG, HDL-c, FBG, hs-CRP, TyG, LM, left circumflex (LCX), RCA, and the number of coronary vessel lesions (all p < 0.05). MACCE patients were generally older with lower LVEF and HDL-c levels, higher TG, FBG, hs-CRP, and TyG values, and had a higher incidence of LM, LCX, RCA, as well as multi-vessel coronary artery disease within the follow-up.

**Table 1:** Baseline demographics and clinical characteristics by the tertiles of the TyG Index

Variable	Total	Tertile 1	Tertile 2	Tertile 3	*P*
N	1140	379	381	380	–
General conditions					
Age, years	59.55 ± 10.98	60.64 ± 11.79	59.76 ± 10.69	58.26 ± 10.32	**0.010**
Sex, male, n (%)	704 (61.8%)	231 (60.9%)	233 (61.2%)	240 (63.2%)	0.787
BMI, kg/m^2^	25.01 ± 3.24	24.08 ± 3.15	25.28 ± 3.24	25.67 ± 3.11	**<0.001**
Heart rate, bpm	69.67 ± 12.42	69.56 ± 12.11	69.65 ± 12.51	69.79 ± 12.67	0.966
SBP, mmHg	125.78 ± 15.43	126.12 ± 16.11	125.66 ± 15.02	125.57 ± 15.16	0.873
DBP, mmHg LVEF, %	75.89 ± 10.48 61.59 ± 8.21	75.56 ± 10.62 61.95 ± 7.68	75.94 ± 9.91 61.54 ± 8.40	76.17 ± 10.90 61.28 ± 8.53	0.719 0.576
Clinical diagnosis					
UAP	830 (72.8%)	283 (74.7%)	282 (74.0%)	265 (69.7%)	0.252
NSTEMI	147 (12.9%)	50 (13.2%)	45 (11.8%)	52 (13.7%)	0.726
STEMI	163 (14.3%)	46 (12.1%)	54 (14.2%)	63 (16.6%)	0.216
Laboratory test					
Creatinine, umol/L	69.62 ± 29.36	67.63 ± 15.55	71.48 ± 46.33	69.87 ± 16.19	0.221
TC, mmol/L	3.96 ± 0.68	3.80 ± 0.67	3.93 ± 0.68	4.15 ± 0.64	**<0.001**
TG, mmol/L	1.47 ± 0.86	0.83 ± 0.20	1.33 ± 0.26	2.24 ± 1.05	**<0.001**
LDL-c, mmol/L	2.28 ± 0.58	2.14 ± 0.56	2.32 ± 0.60	2.38 ± 0.56	**<0.001**
HDL-c, mmol/L	1.15 ± 0.28	1.29 ± 0.31	1.13 ± 0.24	1.03 ± 0.23	**<0.001**
FBG, mmol/L	5.78 ± 1.59	5.26 ± 0.80	5.57 ± 1.04	6.51 ± 2.23	**<0.001**
UA, umol/L	328.31 ± 84.60	303.81 ± 70.64	331.33 ± 82.60	349.79 ± 92.87	**<0.001**
hs-CRP, mg/L	1.20[0.53, 3.17]	0.99[0.42, 3.14]	1.26[0.55, 3.05]	1.30[0.60, 3.44]	**0.025**
CK, U/L	82.00[57.00, 121.25]	84.50[58.00, 126.00]	83.00[57.00, 119.50]	80.00[56.00, 118.00]	0.508
TyG index	8.67 ± 0.53	8.11 ± 0.25	8.65 ± 0.13	9.25 ± 0.33	**<0.001**
Angiography, n (%)					
Left main	64 (5.6%)	19 (5.0%)	24 (6.3%)	21 (5.5%)	0.740
Left anterior descending	830 (72.8%)	272 (71.8%)	284 (74.5%)	274 (72.1%)	0.644
Circumflex	416 (36.5%)	125 (33.0%)	141 (37.0%)	150 (39.5%)	0.172
Right coronary artery	460 (40.4%)	136 (35.9%)	152 (39.9%)	172 (45.3%)	**0.030**
Single-vessel disease	599 (52.5%)	211 (55.7%)	198 (52.0%)	190 (50.0%)	0.283
Multi-vessel disease	474 (41.6%)	139 (36.7%)	163 (42.8%)	172 (45.3%)	**0.047**
Treatment, n (%)					
Operational intervention	846 (74.2%)	246 (64.9%)	287 (75.3%)	313 (82.4%)	**<0.001**
PCI	765 (67.1%)	220 (58.0%)	259 (68.0%)	286 (75.3%)	**<0.001**
CABG	81 (7.1%)	26 (6.9%)	28 (7.3%)	27 (7.1%)	0.966
Medication	294 (25.8%)	133 (35.1%)	94 (24.7%)	67 (17.6%)	**<0.001**
Discharge medication					
Aspirin	1086 (95.3%)	363 (95.8%)	361 (94.8%)	362 (95.3%)	0.801
P_2_Y_12_ inhibitors	992 (87.0%)	318 (83.9%)	323 (84.8%)	351 (92.4%)	**0.001**
Statins	1095 (96.1%)	368 (97.1%)	358 (94.0%)	369 (97.1%)	**0.037**
ACEI/ARBs	114 (10.0%)	32 (8.4%)	35 (9.2%)	47 (12.4%)	0.160
Beta blockers	668 (58.6%)	211 (55.7%)	228 (59.8%)	229 (60.3%)	0.365

Values with p<0.05 in the table have been highlighted in bold

TyG triglyceride glucose index, BMI body mass index, SBP systolic blood pressure, DBP diastolic blood pressure, LVEF left ventricular ejection fraction, UAP unstable angina, NSTEMI non-ST-segment elevation myocardial infarction, STEMI ST-segment elevation myocardial infarction, TC total cholesterol, TG triglyceride, LDL-c low-density lipoprotein cholesterol, HDL-c high-density lipoprotein cholesterol, FBG fasting blood glucose, UA uric acid, hs-CRP high-sensitivity C-reactive protein, CK creatine kinase, PCI percutaneous coronary intervention, CABG coronary artery bypass grafting, ACEI angiotensin-converting enzyme inhibitors, ARBs angiotensin receptor blockers

**Table 2:** Baseline demographics and clinical characteristics of patients with and without MACCE.

Variable	Total	MACCE	Non-MACCE	*P*
N	1140	220	920	–
General conditions				
Age, years	59.55 ± 10.98	62.12 ± 11.28	58.94 ± 10.83	**<0.001**
Sex, male, n (%)	704 (61.8%)	143 (65.0%)	561 (61.0%)	0.270
BMI, kg/m^2^	25.01 ± 3.24	25.22 ± 3.41	24.96 ± 3.19	0.290
Heart rate, bpm	69.67 ± 12.42	70.39 ± 12.73	69.50 ± 12.35	0.340
SBP, mmHg	125.78 ± 15.43	125.99 ± 15.67	125.73 ± 15.38	0.827
DBP, mmHg LVEF, %	75.89 ± 10.48 61.59 ± 8.21	74.99 ± 10.47 59.30 ± 10.08	76.10 ± 10.47 62.14 ± 7.60	0.157 **<0.001**
Clinical diagnosis				
UAP	830 (72.8%)	159 (72.3%)	671 (72.9%)	0.843
NSTEMI	147 (12.9%)	28 (12.7%)	119 (12.9%)	0.934
STEMI	163 (14.3%)	33 (15.0%)	130 (14.1%)	0.741
Laboratory test				
Creatinine, umol/L	69.62 ± 29.36	75.70 ± 62.11	68.29 ± 14.11	0.105
TC, mmol/L	3.96 ± 0.68	3.99 ± 0.67	3.95 ± 0.68	0.428
TG, mmol/L	1.47 ± 0.86	1.64 ± 1.22	1.43 ± 0.75	**0.017**
LDL-c, mmol/L	2.28 ± 0.58	2.29 ± 0.58	2.28 ± 0.58	0.810
HDL-c, mmol/L	1.15 ± 0.28	1.11 ± 0.27	1.16 ± 0.29	**0.013**
FBG, mmol/L	5.78 ± 1.59	6.20 ± 2.27	5.68 ± 1.36	**0.001**
UA, umol/L	328.31 ± 84.60	338.03 ± 91.28	326.01 ± 82.84	0.062
hs-CRP, mg/L	1.20[0.53, 3.17]	1.70[0.65, 3.77]	1.12[0.48, 3.01]	**0.003**
CK, U/L	82.00[57.00, 121.25]	87.00[58.00, 128.50]	81.00[57.00, 121.00]	0.248
TyG index	8.67 ± 0.53	8.80 ± 0.58	8.64 ± 0.51	**<0.001**
Angiography, n (%)				
Left main	64 (5.6%)	23 (10.5%)	41 (4.5%)	**0.001**
Left anterior descending	830 (72.8%)	163 (74.1%)	667 (72.5%)	0.634
Circumflex	416 (36.5%)	94 (42.7%)	322 (35.0%)	**0.032**
Right coronary artery	460 (40.4%)	107 (48.6%)	353 (38.4%)	**0.005**
Single-vessel disease	599 (52.5%)	93 (42.3%)	506 (55.0%)	**0.001**
Multi-vessel disease	474 (41.6%)	116 (52.7%)	358 (38.9%)	**<0.001**
Treatment, n (%)				
Operational intervention	846 (74.2%)	169 (76.8%)	677 (73.6%)	0.313
PCI	765 (67.1%)	155 (70.5%)	610 (66.3%)	0.239
CABG	81 (7.1%)	14 (6.4%)	67 (7.3%)	0.634
Medication	294 (25.8%)	51 (23.2%)	243 (26.4%)	0.325
Discharge medication				
Aspirin	1086 (95.3%)	208 (94.5%)	878 (95.4%)	0.577
P_2_Y_12_ inhibitors	992 (87.0%)	198 (90.0%)	794 (86.3%)	0.143
Statins	1095 (96.1%)	215 (97.7%)	880 (95.7%)	0.156
ACEI/ARBs	114 (10.0%)	27 (12.3%)	87 (9.5%)	0.211
Beta blockers	668 (58.6%)	136 (61.8%)	532 (57.8%)	0.280

Values with p<0.05 in the table have been highlighted in bold

TyG triglyceride glucose index, BMI body mass index, SBP systolic blood pressure, DBP diastolic blood pressure, LVEF left ventricular ejection fraction, UAP unstable angina, NSTEMI non-ST-segment elevation myocardial infarction, STEMI ST-segment elevation myocardial infarction, TC total cholesterol, TG triglyceride, LDL-c low-density lipoprotein cholesterol, HDL-c high-density lipoprotein cholesterol, FBG fasting blood glucose, UA uric acid, hs-CRP high-sensitivity C-reactive protein, CK creatine kinase, PCI percutaneous coronary intervention, CABG coronary artery bypass grafting, ACEI angiotensin-converting enzyme inhibitors, ARBs angiotensin receptor blockers

### Relationship of the TyG index with the long-term prognosis among SMuRF-less ACS patients

Univariate Cox regression analysis was conducted to identify variables associated with MACCE and ischemia-driven revascularization. As shown in Table 3, several factors were independently associated with ischemia-driven revascularization in ACS patients without SMuRFs, including sex, operational intervention, LM lesion, multi-vessel lesion, P_2_Y_12_i, TG, HDL-c, FBG, as well as the TyG index. Moreover, age, LM lesion, multi-vessel lesion, LVEF, creatinine, TG, HDL-c, UA, FBG, and the TyG index were identified as independent risk factors for MACCE among ACS patients without SMuRFs.

**Table 3:** The results of the univariate Cox regression analysis related to ischemia-driven revascularization and MACCE

Variable	ischemia-driven revascularization		MACCE	
	HR (95%CI)	P	HR (95%CI)	P
Age	0.989 (0.973, 1.004)	0.142	1.024 (1.011, 1.037)	** < 0.001**
Sex	0.587 (0.402, 0.858)	**0.006**	0.836 (0.634, 1.103)	0.205
BMI	1.034 (0.982, 1.090)	0.203	1.025 (0.983, 1.069)	0.256
SBP	1.003 (0.992, 1.014)	0.613	1.001 (0.992, 1.010)	0.815
DBP	0.994 (0.978, 1.011)	0.491	0.991 (0.978, 1.003)	0.149
Heart rate	0.995 (0.981, 1.009)	0.489	1.005 (0.995, 1.016)	0.306
Operational intervention	1.666 (1.072, 2.590)	**0.023**	1.197 (0.875, 1.637)	0.261
LM	2.479 (1.448, 4.241)	**0.001**	2.356 (1.529, 3.630)	** < 0.001**
Multi-vessel	1.664 (1.187, 2.333)	**0.003**	1.686 (1.294, 2.197)	** < 0.001**
NSTE-ACS	1.089 (0.663, 1.789)	0.737	0.955 (0.659, 1.382)	0.806
STEMI	0.918 (0.559, 1.509)	0.737	1.048 (0.723, 1.517)	0.806
Aspirin	1.227 (0.502, 2.998)	0.653	0.809 (0.452, 1.449)	0.477
P_2_Y_12_i	2.140 (1.088, 4.209)	**0.027**	1.378 (0.887, 2.141)	0.153
Beta-blocker	1.263 (0.890, 1.794)	0.191	1.166 (0.889, 1.531)	0.268
Statin	1.773 (0.565, 5.568)	0.327	1.732 (0.713, 4.203)	0.225
ACEI/ARB	1.005 (0.567, 1.780)	0.987	1.328 (0.887, 1.986)	0.168
LVEF	0.989 (0.968, 1.010)	0.298	0.968 (0.955, 0.983)	** < 0.001**
hs-CRP	0.994 (0.957, 1.033)	0.770	1.013 (0.986, 1.040)	0.355
Creatinine	1.002 (0.998, 1.006)	0.298	1.003 (1.001, 1.005)	**0.001**
TG	1.289 (1.153, 1.441)	** < 0.001**	1.229 (1.101, 1.371)	** < 0.001**
TC	1.102 (0.857, 1.416)	0.449	1.078 (0.886, 1.312)	0.451
HDL-c	0.485 (0.253, 0.928)	**0.029**	0.536 (0.324, 0.886)	**0.015**
LDL-c	0.988 (0.739, 1.321)	0.934	1.021 (0.812, 1.282)	0.861
UA	1.001 (0.999, 1.003)	0.156	1.002 (1.000, 1.003)	**0.040**
FBG	1.124 (1.047, 1.206)	**0.001**	1.127 (1.066, 1.190)	** < 0.001**
TyG	1.796 (1.322, 2.440)	** < 0.001**	1.645 (1.291, 2.097)	** < 0.001**

Values with p<0.05 in the table have been highlighted in bold

*BMI* body mass index, *SBP* systolic blood pressure, *DBP* diastolic blood pressure, *LM* left main coronary artery, *NSTE-ACS* non-ST-segment elevation acute coronary syndrome, *STEMI ST*-segment elevation myocardial infarction, *ACEI* angiotensin-converting enzyme inhibitors, *ARBs* angiotensin receptor blockers, *LVEF* left ventricular ejection fraction, *hs-CRP* high-sensitivity C-reactive protein, *TG* triglyceride, *TC* total cholesterol, *HDL-c* high-density lipoprotein cholesterol, *LDL-c* low-density lipoprotein cholesterol, *UA* uric acid, *FBG* fasting blood glucose, *TyG* triglyceride glucose index

Table 4 demonstrates that for each SD increase in the TyG index, the HR for MACCE was 1.245 (95% CI: 1.030, 1.504) in Model 3. After adjusting for all relevant factors, subjects in tertile 3 had an HR of 1.693 (95% CI: 1.051, 2.727) for MACCE. There was a statistically significant increasing trend in the risk of MACCE from tertile 1 to tertile 3 (p for trend = 0.020). Similarly, a fully adjusted regression model (Table 5) revealed a strong correlation between the TyG index and revascularization driven by ischemia. According to Model 3, with each SD increase in the TyG index, the risk of ischemia-driven revascularization increased by 30.3% (95% CI 1.026, 1.653). The fully adjusted HR (95% CI) for ischemia-driven revascularization in tertile 3 was 1.855 (0.998, 3.449), but the p value was not significant (p = 0.051). There was a significant rise in the risk of ischemia-driven revascularization from the lowest tertile to the highest tertile, with a statistically significant trend (p for trend = 0.045).

**Table 4:** Multivariable Cox regression analyses for the association between the TyG index and MACCE

TyG Index	Per SD increase	Tertile 1	Tertile 2	Tertile 3	P for trend
Events, n (%)	220 (19.3)	56 (14.8)	66 (17.3)	98 (25.8)	–
	HR (95%CI)	HR (95%CI)	HR (95%CI)	HR (95%CI)	–
Model 1	1.330 (1.162, 1.524)***	Ref.	1.173 (0.810, 1.700)	1.920 (1.362, 2.706) ***	< 0.001
Model 2	1.261 (1.046, 1.521)*	Ref.	1.146 (0.712, 1.845)	1.740 (1.084, 2.794) *	0.015
Model 3	1.245 (1.030, 1.504)*	Ref.	1.102 (0.683, 1.778)	1.693 (1.051, 2.727) *	0.020

Model 1: adjusted for age, sex, and BMI

Model 2: adjusted for Model 1 + SBP, heart rate, LVEF, creatinine, HDL-c, LDL-c, hs-CRP and UA

Model 3: adjusted for Model 2 + LM, Multi-vessel, operational intervention, NSTE-ACS and discharge medication

^*^P < 0.05

^**^P < 0.01

^***^P < 0.001

**Table 5:** Multivariable Cox regression analyses for the association between the TyG index and ischemia-driven revascularization

TyG Index	Per SD increase	Tertile 1	Tertile 2	Tertile 3	P for trend
Events, n (%)	135 (11.8)	33 (8.7)	43 (11.3)	59 (15.5)	–
	HR (95%CI)	HR (95%CI)	HR (95%CI)	HR (95%CI)	–
Model 1	1.361 (1.153, 1.607)***	Ref.	1.327 (0.833, 2.113)	1.881 (1.211, 2.922)**	0.004
Model 2	1.332 (1.052, 1.687)*	Ref.	1.378 (0.746, 2.547)	1.923 (1.037, 3.564)*	0.033
Model 3	1.303 (1.026, 1.653)*	Ref.	1.356 (0.732, 2.512)	1.855 (0.998, 3.449)	0.045

Model 1: adjusted for age, sex, and BMI

Model 2: adjusted for Model 1 + SBP, heart rate, LVEF, creatinine, HDL-c, LDL-c, hs-CRP and UA

Model 3: adjusted for Model 2 + LM, Multi-vessel, operational intervention, NSTE-ACS and discharge medication

^*^P < 0.05

^**^P < 0.01

^***^P < 0.001

**Fig. 2:**
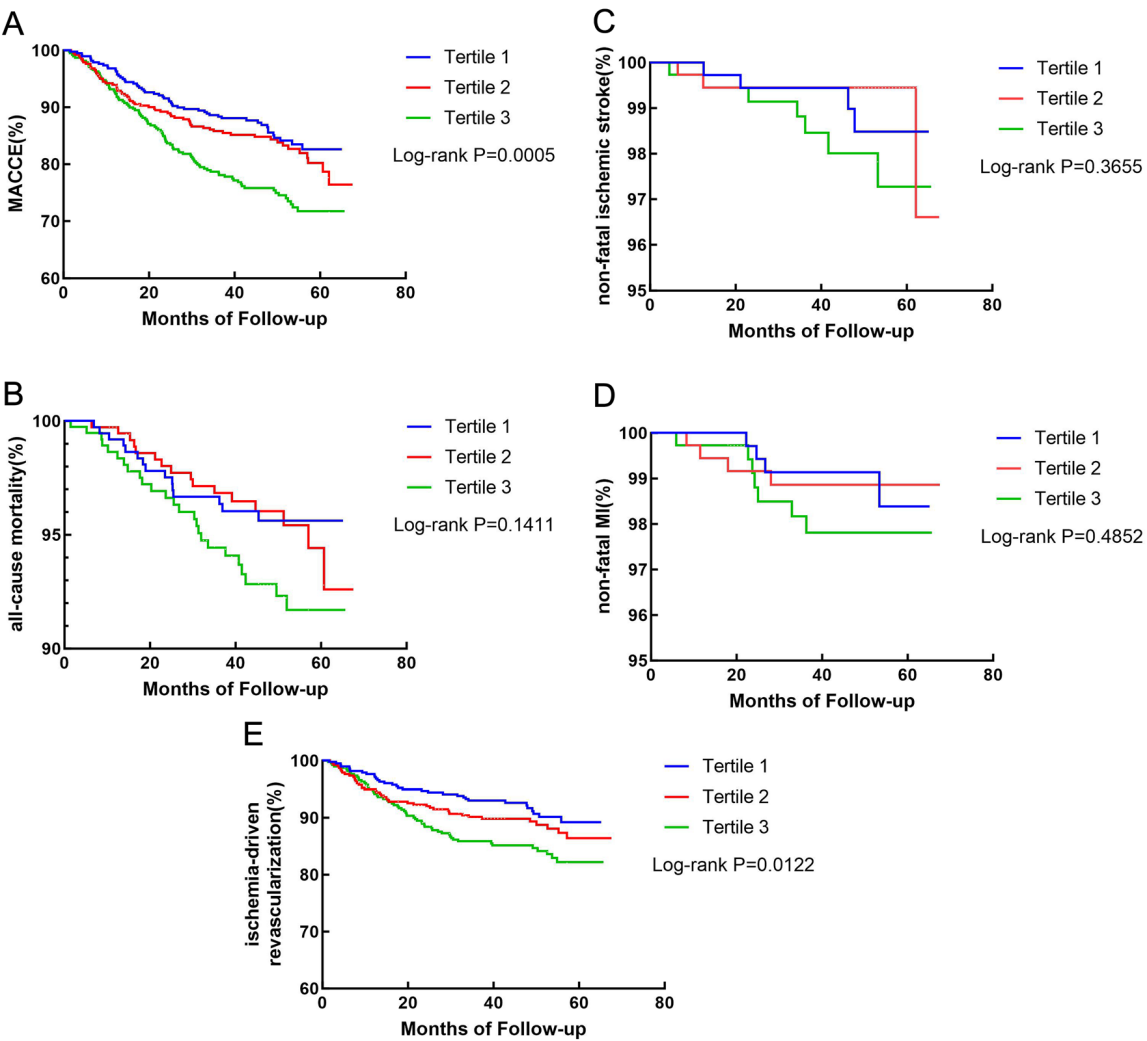
Kaplan–Meier survival curves according to the tertiles of the TyG index. **A** Kaplan–Meier survival curve of MACCE; **B** Kaplan–Meier survival curve of all-cause mortality; **C** Kaplan–Meier survival curve of non-fatal ischemic stroke; **D** Kaplan–Meier survival curve of non-fatal MI; **E** Kaplan–Meier survival curve of ischemia-driven revascularization. *TyG* triglyceride glucose index, *MACCE* major adverse cardiac and cerebrovascular events, *MI* myocardial infarction

The Kaplan–Meier method was used to evaluate the combined incidence of MACCE and its components over time in the three groups. A higher TyG index was associated with an increased cumulative incidence of MACCE (Fig. 2A, log-rank p = 0.0005) and ischemia-driven revascularization (Fig. 2E, log-rank p = 0.0122). The cumulative incidences of all-cause mortality (Fig. 2B, log-rank test, p = 0.1411), non-fatal ischemic stroke (Fig. 2C, log-rank test, p = 0.3655), and non-fatal MI (Fig. 2D, log-rank test, p = 0.4852) were not significantly different among the three clusters.

### Subgroup analysis

**Fig. 3:**
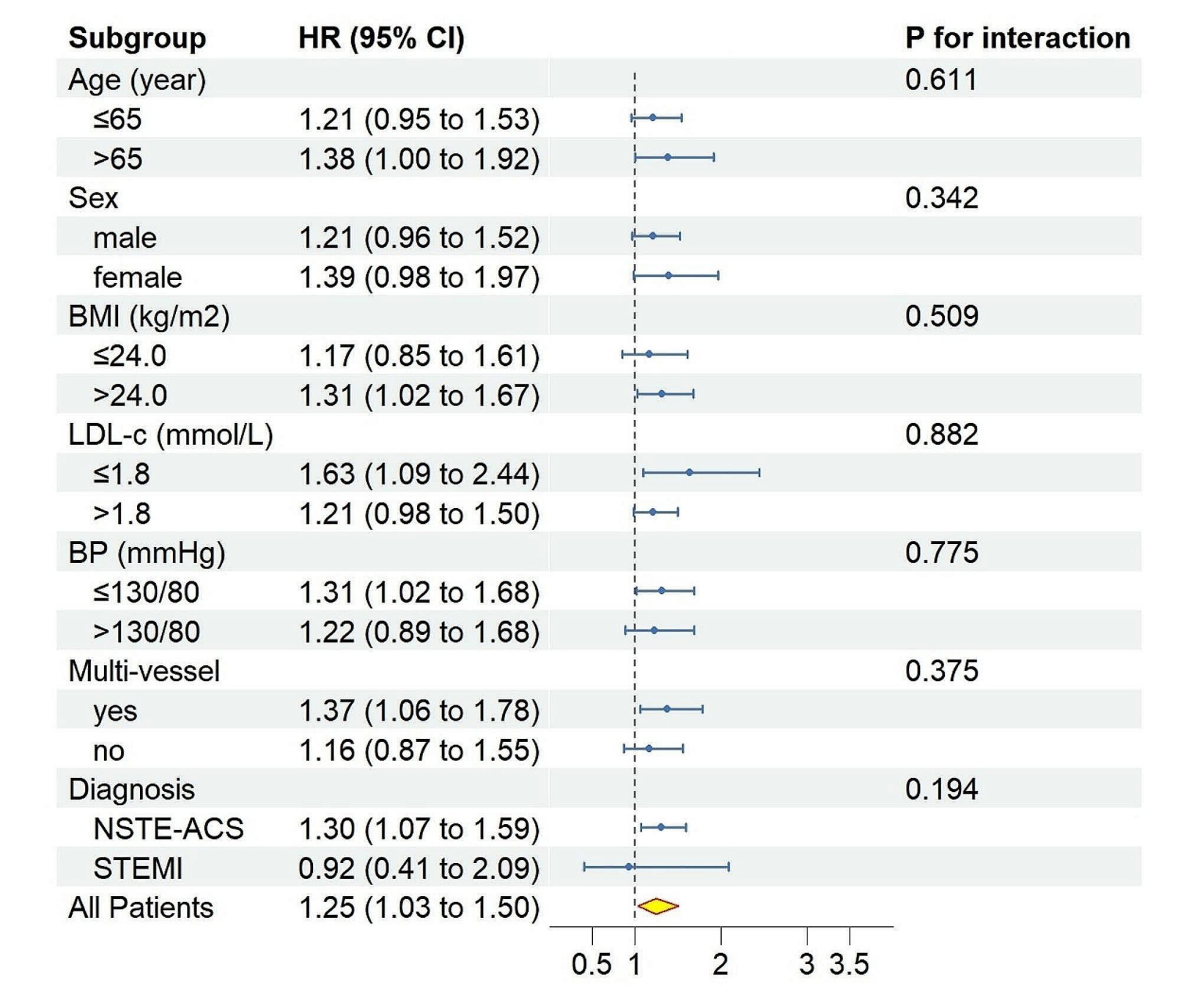
Forest plot of MACCE according to different subgroups. Adjusted model included age, sex, TyG index, body mass index, systolic blood pressure, heart rate, LVEF, creatinine, HDL-c, LDL-c, hs-CRP, uric acid, LM, Multi-vessel lesion, operational intervention, NSTE-ACS, aspirin, P_2_Y_12_ inhibitors, statins, ACEI/ARBs, Beta blockers. HR was evaluated by per SD increase of TyG

Figure 3 displays the results, with stratified analysis showing no variation in the predictive capacity of the TyG index for MACCE based on age (≤ 65 or > 65 years), sex (male/female), BMI (≤ 24.0 or > 24.0 kg/m^2^), LDL-c (≤ 1.80 or > 1.80 mmol/L), blood pressure (≤ 130/80 or > 130/80 mmHg), multi-vessel lesion (yes/no), or diagnosis (NSTE-ACS/STEMI) (for all, p for interaction > 0.05).

## Discussion

This research has shown, for the first time, a connection of the TyG index with the occurrence of MACCE in ACS patients without SMuRFs. Our study included 1140 SMuRF-less ACS patients, with a median follow-up period of four years. During this time, the overall incidence of MACCE was 19.3%. The primary results of our research included: (1) The risk of cardiovascular adverse events in SMuRF-less ACS patients significantly increased with an elevation in the TyG index, primarily due to ischemia-driven revascularization; (2) The TyG index had a significant impact on ischemia-driven revascularization and the rate of MACCE. Univariate regression analysis indicated that an increase in the TyG index significantly elevated the rates of revascularization and MACCE; (3) Following the adjustment for potential confounding factors, the TyG index, whether considered as a continuous or categorical variable, was independently associated with an increase in MACCE; (4) As a reliable indicator of IR, the TyG index may hold significant clinical value for early risk stratification in SMuRF-less ACS patients.

Our findings have confirmed that patients with a higher TyG index were more susceptible to multi-vessel disease, consistent with results reported in the literature [20, 28]. Furthermore, we have identified a correlation of a high TyG index with an increased rate of ischemia-driven revascularization, suggesting that in the ACS population, a high TyG index could effectively predict the need for revascularization [29]. Previous studies have also established a significant link of the TyG index with traditional risk factors for coronary heart disease [20, 30]. These conventional risk factors, along with the TyG index, have a close link with the prognosis of the ACS population. Besides, in patients with chronic coronary syndrome (CCS), a study conducted by Otsuka et al. revealed that individuals without diabetes, but with elevated TyG index, were at an increased risk of major adverse cardiac events (MACE) [31]. The study suggests that an elevated TyG index serves as a valuable marker for identifying patients with prediabetes who are at high risk of MACE in patients with CCS. However, the prognostic impact of the TyG index on the ACS population without standard modifiable cardiovascular risk factors such as diabetes remains unclear. Regarding of the potential prognostic significance of the TyG index, it is crucial to assess its predictive ability specifically for this subgroup of ACS patients. It will not only expand the clinical use of the TyG index but also aid in the early detection and treatment of the SMuRF-less ACS population. Our study, focusing on SMuRF-less ACS patients, brought significant innovation and clinical guidance.

Currently, an increasing number of studies have identified a rising trend in ACS patients without SMuRFs [7, 32], which has significantly impacted the field of cardiovascular medicine. The presence of these patients suggests that there may be other, as yet unrecognized, risk factors or biomarkers involved in the development and progression of CVD. Research has shown that these patients experience worse short-term and long-term clinical outcomes compared to patients with at least one SMuRF [7]. It poses a significant challenge to current prevention and treatment strategies for CVD. SMuRF-less ACS patients form a distinct category within the ACS cohort, lacking SMuRFs, yet their prognosis is relatively poor. Additionally, a study by Mazhar et al. found that patients with clinical coronary atherosclerosis without SMuRFs exhibit similar rates of plaque progression to those with traditional risk factors [33], suggesting the presence of yet undiscovered factors that could promote plaque progression. Furthermore, nonstandard risk factors such as inflammation, oxidative stress and coagulation activation may play a role in the progression of atherosclerosis [34–36]. IR is a condition characterized by a disturbance in the absorption and use of glucose, resulting in decreased sensitivity to insulin. It leads to abnormal fluctuations in blood glucose and lipid levels, including elevated plasma glucose and TC levels, along with reduced HDL-c levels [37]. The TyG index, derived from TG and FBG, is considered a reliable, practical, and cost-effective tool for assessing IR based on this theoretical foundation [38]. The TyG index not only excels in assessing IR but is also closely associated with future cardiovascular events, serving as an independent risk factor for ACS patients [39, 40]. Moreover, previous studies have indicated that the ACS population with a high TyG index has a higher risk of major adverse cardiovascular events [41, 42]. We hypothesize that the TyG index can similarly predict the risk of adverse cardiovascular events in SMuRF-less ACS patients. According to our findings, it could be inferred that the TyG index is an independent predictor of MACCE in SMuRF-less ACS patients.

In summary, our study revealed that the TyG index was closely related to MACCE in ACS patients without SMuRFs, and it served as a reliable indicator for evaluating the poor prognosis of such patients. Although the exact mechanisms underlying the poor prognosis of these patients remain unclear, we speculate that it may be related to potential risk factors, such as IR, in SMuRF-less ACS patients. Previous research has shown that IR not only disrupted insulin signaling in vital intimal cells such as vascular smooth muscle cells and macrophages, but also created a proinflammatory environment and dyslipidemia. These factors ultimately contributed to the progression of plaque formation [43]. IR has also been found to be associated with elevated levels of free fatty acids and increased accumulation of dietary fat, resulting in lipotoxicity. This, in turn, contributes to the advancement of atherosclerosis [44]. Furthermore, IR has shown a significant correlation with CVD, independent of hyperglycemia [45]. This finding suggests that IR may participate in the pathogenesis of CVD in individuals with ACS but without SMuRFs, warranting further exploration in subsequent studies.

Coronary artery plaque burden is a significant risk factor for plaque rupture and a primary cause of ACS. A coronary computed tomography angiography (CCTA) study conducted by Yamaura et al. discovered that in patients without overt known CAD, three-vessel low-attenuation non-calcified coronary plaque (LAP) volume is an important predictor of the primary endpoints, independent of CAD-RADS ≥ 3 (severe stenosis and total occlusion) [46]. Assessing coronary artery calcium score (CACS) and epicardial adipose tissue volume (EAV) through CCTA offers a non-invasive method to identify patients with increased LAP volume that can lead to coronary events. Vascular inflammation is a key component of the pathophysiology of atherosclerosis, associated with the risk of plaque rupture and the occurrence of ACS. Pericoronary adipose tissue attenuation (PATA) is a novel CT parameter that reflects vascular inflammatory activity [47]. Compared with morphological CT features associated with unstable plaques, e.g. spotty calcification, its advantage lies in its ability to detect earlier, thus making it more sensitive for diagnosing vulnerable plaques. PATA has been associated with prognosis, predicting cardiac and all-cause mortality, with incremental value to age, gender, cardiovascular risk factors, and CT calcium score [47]. In conclusion, inflammation may explain the elevated risk of future cardiovascular events in this population. Our research results further enhanced the risk assessment and management of the SMuRF-less ACS population, thereby promoting the overall prevention of CVD.

## Limitations

There were several limitations in our study. Firstly, this was a single-center, retrospective, observational analysis. Thus, it was not feasible to establish causality based on the findings alone. Future investigations, preferably prospective and multicenter studies, are necessary to further validate the results obtained in this study. Secondly, the sample size of this study was limited, and despite adjusting for various confounding factors, it was impossible to completely eliminate data bias. Thirdly, we only included baseline TyG index data without dynamically observing changes in the TyG index, which could potentially influence the prognosis. Lastly, all the subjects in this study were from China, which may restrict the generalizability of our findings to other ethnic groups.

## Conclusion

This study demonstrated a significant correlation between the TyG index, calculated based on blood glucose levels after the acute admission, and adverse cardiovascular outcomes in SMuRF-less ACS patients, indicating the potential of the TyG index as a reliable tool for predicting cardiovascular events, thereby contributing to the development of risk assessment tools for SMuRF-less ACS patients.

## Data Availability

The datasets used/or analyzed during the current study are available from the corresponding author on reasonable request.

## Abbreviations

CVD: Cardiovascular disease
CAD: Coronary artery disease
SMuRFs: Standard modifiable cardiovascular risk factors
ACS: Acute coronary syndrome
TyG index: Triglyceride-glucose index
IR: Insulin resistance
TC: Total cholesterol
LDL-c: Low-density lipoprotein cholesterol
BMI: Body mass index
LVEF: Left ventricular ejection fraction
UAP: Unstable angina pectoris
NSTEMI: Non-ST-segment elevation myocardial infarction
NSTE-ACS: Non-ST-segment elevation acute coronary syndrome
STEMI: ST-segment elevation myocardial infarction
CAG: Coronary angiography
MACCE: Major adverse cardiac and cerebrovascular events
MI: Myocardial infarction
HR: Hazard ratio
SBP: Systolic blood pressure
DBP: Diastolic blood pressure
HDL-c: High-density lipoprotein cholesterol
UA: Uric acid
LM: Left main coronary artery
LCX: Left circumflex artery
RCA: Right coronary artery
PCI: Percutaneous coronary intervention
CABG: Coronary artery bypass graft
CI: Confidence interval
TG: Triglyceride
FBG: Fasting blood glucose
hs-CRP: High-sensitivity C-reactive protein
CK: Creatine kinase
ACEI: Angiotensin-converting enzyme inhibitors
ARBs: Angiotensin receptor blockers
HbA1c: Glycosylated hemoglobin
CT: Computed tomography
CCS: Chronic coronary syndrome
MACE: Major adverse cardiac events
CCTA: Coronary computed tomography angiography
LAP: Low-attenuation non-calcified coronary plaque
CACS: Coronary artery calcium score
EAV: Epicardial adipose tissue volume
PATA: Pericoronary adipose tissue attenuation
SD: Standard deviation
IQR: Interquartile range
